# Michigan

**Published:** 1897-03

**Authors:** 


*Michigan* has been a great sufferer the past year from liver-fluke, hydatids in the brain, tapeworm, and stomach-worms, owing to the wet condition of much of the pasture-land. More care in using these lands in wet seasons will better control the severe losses incidental to these diseased conditions until more light is shed upon the means of destroying parasites within and without the animal body.

